# Transmission and pathogenicity of novel reassortants derived from Eurasian avian-like and 2009 pandemic H1N1 influenza viruses in mice and guinea pigs

**DOI:** 10.1038/srep27067

**Published:** 2016-06-02

**Authors:** Weili Kong, Qinfang Liu, Yipeng Sun, Yu Wang, Huijie Gao, Lirong Liu, Zhihua Qin, Qiming He, Honglei Sun, Juan Pu, Dayan Wang, Xin Guo, Hanchun Yang, Kin-Chow Chang, Yuelong Shu, Jinhua Liu

**Affiliations:** 1 Key Laboratory of Animal Epidemiology and Zoonosis, Ministry of Agriculture, College of Veterinary Medicine, and State Key Laboratory of Agrobiotechnology, China Agricultural University, Beijing 100193, China; 2 Department of Avian Infectious Disease, Shanghai Veterinary Research Institute, Chinese Academy of Agricultural Sciences, Shanghai, Innovation Team for Pathogen Ecology Research on Animal Influenza Virus, Shanghai 200241 China; 3 National Institute for Viral Disease Control and Prevention, China CDC, Key Laboratory for Medical Virology, National Health and Family Planning Commission, Beijing 102206, China; 4 School of Veterinary Medicine and Science, University of Nottingham, Sutton Bonington Campus, Loughborough, LE12 5RD, United Kingdom

## Abstract

Given the present extensive co-circulation in pigs of Eurasian avian-like (EA) swine H1N1 and 2009 pandemic (pdm/09) H1N1 viruses, reassortment between them is highly plausible but largely uncharacterized. Here, experimentally co-infected pigs with a representative EA virus and a pdm/09 virus yielded 55 novel reassortant viruses that could be categorized into 17 genotypes from Gt1 to Gt17 based on segment segregation. Majority of novel reassortants were isolated from the lower respiratory tract. Most of reassortant viruses were more pathogenic and contagious than the parental EA viruses in mice and guinea pigs. The most transmissible reassortant genotypes demonstrated in guinea pigs (Gt2, Gt3, Gt7, Gt10 and Gt13) were also the most lethal in mice. Notably, nearly all these highly virulent reassortants (all except Gt13) were characterized with possession of EA H1 and full complement of pdm/09 ribonucleoprotein genes. Compositionally, we demonstrated that EA H1-222G contributed to virulence by its ability to bind avian-type sialic acid receptors, and that pdm/09 RNP conferred the most robust polymerase activity to reassortants. The present study revealed high reassortment compatibility between EA and pdm/09 viruses in pigs, which could give rise to progeny reassortant viruses with enhanced virulence and transmissibility in mice and guinea pig models.

Influenza is one of the most important causes of respiratory tract infection that results in around 200,000–500,000 deaths each year worldwide. In influenza pandemics, incidence and pneumonic manifestations are often much more frequent with ensuing surge in global mortality. Historically, pandemic influenza A viruses, such as from 1957, 1968 and 2009, were all reassortants derived from human and animal influenza viruses^1^,^2^. Pigs are thought to be “mixing vessels” for influenza viruses and play a significant role in the generation of pandemic viruses by genetic reassortment owing to their permissiveness to both avian and human influenza viruses^3^. The most recent 2009 H1N1 pandemic was caused by a novel reassortant swine H1N1 virus with genetic segments from Eurasian avian-like and North American swine influenza viruses (SIVs)^2^ which is a stark reminder of the need to continually monitor the evolving nature of influenza viruses in pigs in anticipation for the next pandemic.

Different lineages of influenza viruses, including Eurasian avian-like (EA) H1N1, triple reassortant (TR) H1N2, classical swine H1N1, 2009 pdm H1N1 and H3N2, are co-circulating in pig herds with some geographic restrictions^4^,^5^,^6^. EA H1N1 virus first emerged in Europe in 1979 and has replaced the classical swine H1N1 virus which indicates that the EA virus has competitive advantage in pigs^7^,^8^,^9^. In 2007, EA virus was first reported in Hong Kong and eventually became dominant in China^10^,^11^. The EA virus has been documented to cause respiratory infection in humans^12^,^13^. Since the global human population is expected to have little to no immunity against EA virus or virus with EA HA gene^14^,^15^,^16^,^17^, the EA swine lineage of viruses could be a potential genetic source for the development of future pandemic strains.

The pdm/09 virus continues to circulate in humans and has effectively become a seasonal virus. Pigs are highly susceptible to pdm/09 virus, which repeatedly infected pigs around the world and reassortment with pdm/09 virus has also occurred with many other SIV lineages apart from the EA lineage^18^,^19^. Previous studies have identified reassortant swine influenza virus derived from EA and pdm/09 lineages in Hong Kong, Germany, Italy and United Kingdom^20^,^21^,^22^,^23^,^24^,^25^. Recent surveys of SIVs from 2009–2012 in South China found marked changes in the evolutionary patterns and ecosystem of SIVs and that only the internal genes of the pdm/09 virus were present in dominant lineages in pigs^10^. Collectively, these observations suggest that reassortants between pdm/09 and EA H1N1 viruses could potentially generate reassortant viruses with novel antigenicity for humans. Although many reassortants between pdm/09-origin viruses and enzootic SIVs have been detected, no systematic study has been reported on reassortants between pdm/09 and EA viruses.

To better understand the genetic diversity and biological properties of novel reassortants between pdm/09 and EA influenza virus, we experimentally co-infected pigs with EA swine H1N1 and pdm/09 viruses, and examined the reassortant patterns of progeny viruses and their pathogenicity/transmissibility in mice and guinea pigs. We found that the two virus lineages could generate reassortant viruses with enhanced pathogenicity and transmissibility in both animal models, and that the most pathogenic and transmissible reassortants retained the EA H1 and full complement of pdm/09 RNP gene segments.

## Results

### Reassortants from co-infection of pigs with parental EA/FJ04 and pdm/BJ09 H1N1 viruses

To systematically examine reassortants obtained from co-infection of swine with EA and pdm/09 viruses, a representative EA virus A/Swine/Fujian/204/2007 (EA/FJ04) and a representative pdm/09 virus A/Beijing/7/2009 (pdm/BJ09) were selected for coinfection of six pigs inoculated by intranasal and intratracheal routes. At 3 days post-infection (dpi), all pigs exhibited clinical symptoms including coughing and pyrexia. Daily nasal washes from 1 to 5 dpi, and bronchoalveolar fluids (BALFs) from 3 euthanatized pigs each time at 3 and 5 dpi were collected. Ten plaque purified viruses were isolated and sequenced from each nasal wash and BALF sample. A total of 300 purified viruses (about 50 per pig) were isolated, of which 240 viruses were from nasal washes and 60 viruses were from BALFs. Based on the parental origin of eight gene segments, plaque-purified viruses were classified as EA/FJ04 or pdm/BJ09 genotype if they contained all eight genes from only one parental virus, and as reassortants if they contained segments of both parental viruses.

Pdm/BJ09 virus was most frequently isolated from all six pigs (221/240 from nasal passage and 24/60 from low respiratory tract, LRT). In contrast, EA/FJ04 virus was not found in any of the samples. A total of 55 reassortants identified from the 300 virus clones (55/300, 18%). Sixty percent (33/55) of all reassortant viruses possessed the EA/FJ04 H1 gene, which in turn could be assigned into 10 distinct genotypes, Gt1 to Gt10, based on segment segregation patterns (Table 1). The remaining 40% (22/55) of reassortants housed the pdm/BJ09 H1 gene, which could be designated into 7 distinct genotypes: Gt11 to Gt17 (Table 1). Gt2, mainly found in BALFs, was the most common genotype constituting more than 25% of all reassortants (14/55) where the H1 and NS segments were from the parental EA/FJ04 virus with the remaining 6 gene segments from the pdm/BJ09 virus (Table 1). Reassortants Gt13 at 13% (7/55) and Gt14 also at 13% (7/55) were the dominant pdm/BJ09 H1 genotypes isolated only from the nasal passage.

Remarkably, all 55 reassortant viruses carried the PB2, PA, and NP from pdm/BJ09 virus. On the other hand, the NS and HA genes of EA/FJ04 virus were highly represented in the reassortants at 65% (36/55) and 60% (33/55) respectively. The EA/FJ04 virus PB1, NA, M gene segments were found in only 25%, 35% and 18% of all reassortants respectively. In summary, the most frequent reassortant genotypes, based on H1 parental EA/FJ04 classification of Gt1 to Gt10 or H1 pdm/BJ09 of Gt11 to Gt17, were Gt2 (25%), Gt13 (13%), Gt14 (13%) and Gt7 (5/55, 9%). The four genotypes all contained RNP and M gene segments derived from the pdm/BJ09 virus which suggest that these internal genes have competitive advantage in replication over corresponding EA/FJ04 genes (Table 1).

### Distribution of reassortant viruses in respiratory tracts of co-infected pigs

There is clear differential expression of avian-like sialic acid α2,3-galactose (SAα2,3-Gal) linked and human-like sialic acid α2,6-galactose (SAα2,6-Gal) linked receptors along the porcine respiratory tract, with SAα2,3-Gal linked receptors mainly found in the LRT^26^. We found that the frequency of reassortment was low in nasal passage throughout the infection period, but progressively increased during infection in lungs (Fig. S1). Thirty-six out of 60 (60%) viruses isolated from BALFs and 19 out of 240 (8%) from nasal washes were reassortant viruses. The 19 reassortants isolated from the nasal passage comprised five genotypes of Gt7, Gt10, Gt13, Gt14, and Gt15 (Table 1). Eighty four percent (16/19) of nasal passage-derived reassortants comprised H1 of parental pdm/BJ09 virus. Only 3 of the 19 reassortant viruses, one Gt7 and two Gt10 viruses carried the H1 of EA/FJ04 virus. The 36 reassortant viruses isolated from the LRT comprised 13 different genotypes excluding Gt10, Gt13, Gt14 and Gt15. Seventy eight percent (28/36) of these reassortants carried the EA/FJ04 H1 gene. Of note, Gt7 was found in both upper and LRT. In summary, majority of the reassortants harboring the H1 from the EA/FJ04 virus were observed in the LRT of infected pigs, coincident with the presence of avian-like sialic acid receptors^26^.

### Transmissibility of EA/FJ04 and pdm/BJ09 reassortants in guinea pigs

**Figure 1:**
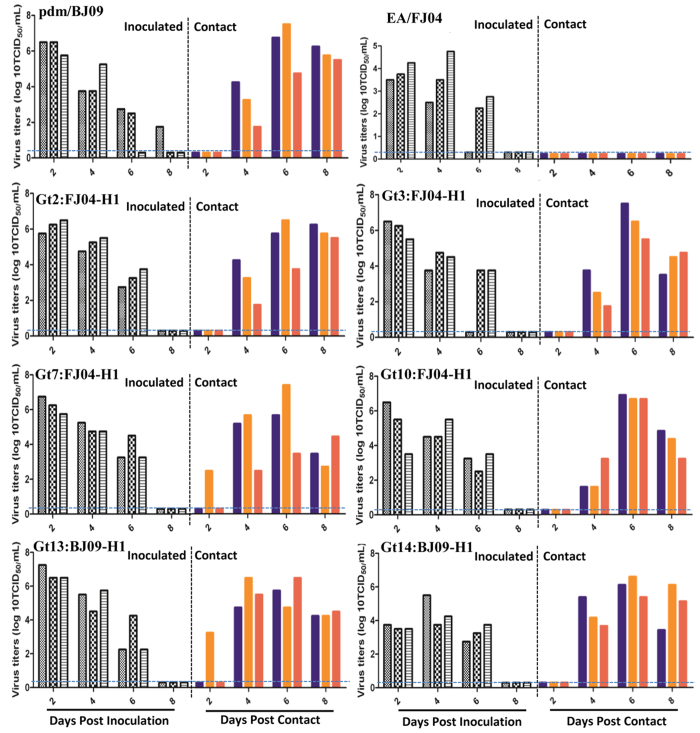
Replication and transmission of reassortants Gt2, 3, 7, 10, 13 and 14 (transmission group I) and parental pdm/BJ09 and EA/FJ04 viruses in guinea pigs. Each group of three guinea pigs (grey columns) was intranasally (i.n.) inoculated with 10^5^TCID_50_ of each virus; 24 hours later, three in-contact guinea pigs (colorful columns) were placed with each infected group. Nasal washes were collected every two days from all animals. Each bar represents the virus titer from nasal wash of an individual animal. The dashed blue lines indicate the lower limit of detection.

**Figure 2:**
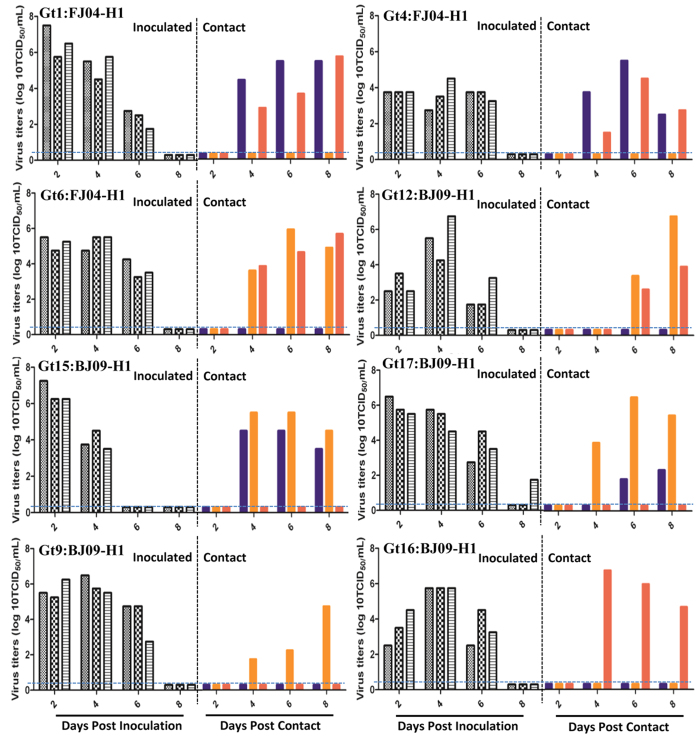
Replication and transmission of reassortants (Gt1, 4, 6, 9, 12, 15, 16 and 17) of higher transmissibility (transmission group II) than the parent EA/FJ04 virus in guinea pigs. Each group of three guinea pigs (grey columns) was i.n. inoculated with 10^5^TCID_50_ of each virus; 24 hours later, three in-contact guinea pigs (colorful columns) were placed with each group. Nasal washes were collected every two days from all animals. Each bar represents the virus titer from nasal wash of an individual animal. The dashed blue lines indicate the lower limit of detection.

Guinea pigs were used to determine the transmissibility of reassortant viruses. As shown in Fig. 1, both parental EA/FJ04 and pdm/BJ09 viruses readily infected guinea pigs. Pdm/BJ09 virus replicated more efficiently in the nasal passage than EA/FJ04 virus and was detected in the nasal washes of all three inoculated and three in-contact guinea pigs, which indicated efficient horizontal transmission. However, there was no transmission of EA/FJ04 virus to in-contact guinea pigs. Based on virus transmissibility, the 17 reassortant genotypes were categorized into three groups:

1. Transmissibility group I comprised 6 reassortant genotypes, Gt2, Gt3, Gt7, Gt10, Gt13 and Gt14, with high transmissibility that was similar to that of the parental pdm/BJ09 virus (Fig. 1). The six genotypes made up 67% (37/55) of all reassortant isolates. Most of the reassortants of Gt2, Gt3, Gt7, and Gt10 in this group harbored EA/FJ04 H1 gene. Notably, all viruses from this group contained PB2, PB1, PA and NP segments of the pdm/BJ09 virus.
2. Transmissibility group II composed of 8 reassortant genotypes, Gt1, Gt4, Gt6, Gt9, Gt12, Gt15, Gt16 and Gt17, that showed reduced transmissibility but still higher than the parental EA/FJ04 virus. Gt1, Gt4, Gt6, Gt12, Gt15 and Gt17 reassortants were transmitted to two out of the three in-contact guinea pigs, while Gt9 and Gt16 reassortants infected one out of three in-contact animals (Fig. 2). The 8 genotypes comprised 20% (11/55) of reassortant viruses. Interestingly, all viruses in this group except Gt1 contained PB2, PA and NP gene segments of the pdm/BJ09 virus and PB1 segment of the EA/FJ04 virus.
3. Transmissibility group III comprised 3 reassortant genotypes, Gt5, Gt8 and Gt11, which showed no horizontal transmission which was similar to that of the parental EA/FJ04 virus. These viruses were only detected in the nasal washes of all three inoculated guinea pigs (Fig. S2). The two genotypes (Gt8, Gt11) harbored PB2, PA and NP gene segments of the pdm/BJ09 virus and PB1 segment of the EA/FJ04 virus.

Seroconversion occurred in all inoculated animals and in infected in-contact animals. For statistical analyses, the area under the nasal wash curve between 2 and 6 dpi (AUC_2–6 dpi_), which measured duration of peak virus shedding, was calculated for each guinea pig, as previously described^27^. The AUC_2–6 dpi_ value of the EA/FJ04 virus group was significantly lower than that of the pdm/BJ09 virus group and all other reassortant genotypes (Fig. S3). These results suggested that in guinea pigs pdm/BJ09 genes enhanced replication of reassortant viruses.

### Pathogenicity of EA/FJ04 and pdm/BJ09 reassortants in mice

**Figure 3:**
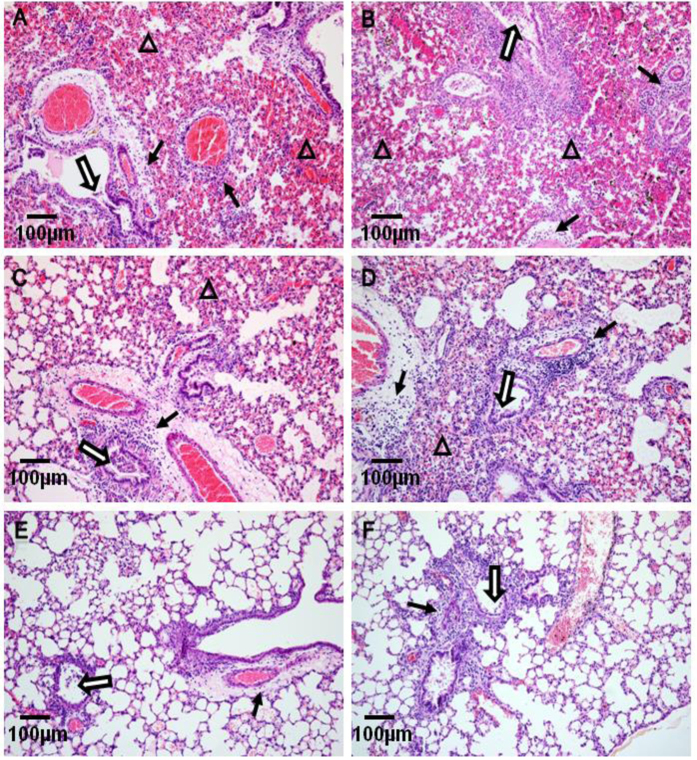
Reassortants (Gt7, 12, 13 and 17) of EA/FJ04 and pdm/BJ09 viruses exhibited a spectrum of lung histopathology at 4 days post infection. (**A**) Gt7 and (**B**) Gt13 viruses caused severe bronchopneumonia and interstitial pneumonia with erosion of epithelial lining (open arrow), widespread infiltrate of monocytic cells (triangle) and interstitial edema around blood vessels (solid arrow). (**C**) Parental pdm/BJ09 and (**D**) Gt17 virus infection resulted in less severe bronchopneumonia with edema, erosion of epithelial lining (open arrow) and some infiltration of monocytic cells (triangle) around blood vessels (solid arrow). (**E**) Parental EA/FJ04 and (**F**) Gt12 viral infections were least severe with reduced erosion of epithelial cells (open arrow) and few monocytic inflammatory cells (triangle) around blood vessels (solid arrow). Lung sections were stained with hematoxylin and eosin. Scale bar = 100 μm.

All reassortant genotypes and the two parental EA/FJ04 and pdm/BJ09 viruses were assessed for pathogenicity in BALB/c mice. Both parental viruses readily infected mice, and LD_50_ of EA/FJ04 and pdm/BJ09 was >10^5.5^ and 10^4.5^ TCID_50_ respectively. Pdm/BJ09 virus replicated more efficiently in the LRT than EA/FJ04 virus on day 4 following intranasal virus inoculation at 10^5^ TCID_50_. Mice that lost more than 25% of their body weight were euthanized. Pdm/BJ09 virus caused >25% weight loss which ensured complete mortality in mice whereas EA/FJ04 virus caused only 1% weight loss with no mortality. All tested reassortants readily infected mice and replicated in the LRT (Table 2). The reassortant genotypes were additionally categorized into three groups according to their relative pathogenicity in mice compared with parental viruses:

1. Pathogenicity group I comprised 6 reassortant genotypes, Gt2, Gt3, Gt5, Gt7, Gt10 and Gt13, with pathogenicity higher than either parental virus (Table 2). With LD_50_ at10^3.5^ to10^3.8^ TCID_50_ and mean survival time (MST) of 4 to 5.3 days, they constituted 58% (32/55) of reassortant viruses. Infected mice displayed obvious clinical signs of huddling, ruffled fur, severe dyspnea by 4 dpi and complete mortality after 5 days. With this group of reassortants, even at a reduced infection dose of 10^4^ TCID_50_, severe clinical signs were evident (data not shown). Hematoxylin and eosin (H&E) lung histopathology performed on three mice from each genotype at 4 dpi revealed severe bronchopneumonia and interstitial pneumonia, characterized by edema, epithelial erosion and massive infiltration of inflammatory monocytic cells (Fig. 3A,B).
2. Pathogenicity group II composed of 8 reassortant genotypes, Gt4, Gt6, Gt8, Gt11, Gt14, Gt15, Gt16 and Gt17, that showed pathogenicity similar to that of the pdm/BJ09 virus. With LD_50_ at 10^4.3^ to 10^4.5^ TCID_50_ and MST between 4.3–8.3 days, this group of reassortants constituted 36% (20/55) of all reassortant isolates. Infected mice, as in pathogenicity group I, showed severe clinical signs, bronchopneumonia and eventual deaths similar to those caused by the parental pdm/BJ09 virus (Fig. 3C,D). However, this group of reassortants at a lower inoculation dose of 10^4^TCID_50_ elicited no clinical signs in mice (data not shown).
3. Pathogenicity group III comprised 3 reassortant genotypes, Gt1, Gt9 and Gt12, which showed reduced virulence similar to that of the EA/FJ04 virus. This group did not cause any death over 2 weeks of infection and constituted only 5% (3/55) of total reassortant viruses. Clinical signs from infected mice were not apparent and lung histopathological changes were mild to moderate (Fig. 3E,F). Notably, EA/FJ04 virus grew to lower virus titers in lungs at 4 dpi than all reassortants, which suggested that introduction of pdm/BJ09 genes into EA/FJ04 virus enhanced viral replication in mice (Table 2).

In summary, five out of six reassortant genotypes in the most pathogenic category (Gt2, Gt3, Gt5, Gt7 and Gt10 in pathogenicity group I) housed the H1 gene from the EA/FJ04 virus; in particular Gt7 reassortant viruses only contained H1 gene from EA/FJ04, indicating that EA H1 contributed to virulence in these reassortants. All six genotypes in this high pathogenicity group housed the 4 RNP genes of PB1, PB2, PA and NP from the pdm/BJ09 virus, suggesting that pdm/BJ09 RNP is critical to enhanced pathogenicity. Notably, highly pathogenic Gt2, Gt3, Gt7, Gt10 and Gt13 reassortants were earlier found to be highly transmissible in guinea pigs (Fig. 1).

### HA-222G was critical in the pathogenicity of EA H1 reassortant virus in mice

**Figure 4:**
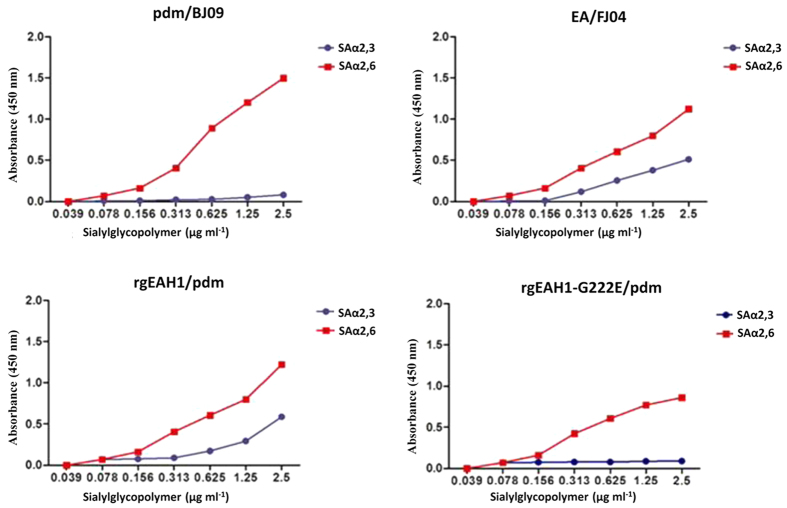
Relative viral affinity for α-2,6-linked and α-2,3-linked sialic acids. Parental pdm/BJ09, parental EA/FJ04, rgEAH1/pdm and rgEAH1-G222E/pdm were subjected to direct binding to sialylglycopolymers containing either α-2,3-linked (blue) or α-2,6-linked (red) sialic acids.

Our above results suggested that the EA/FJ04 HA gene is an important virulence factor in the reassortant viruses. Receptor-binding specificity of HA is an important molecular determinant of host range, transmissibility and replication^28^. To ascertain the receptor-binding properties of the two parental viruses, we performed binding of EA/FJ04 and pdm/BJ09 viruses to a glycan array panel of SAα2,3- or SAα2,6-linked sialosides (Fig. 4). In keeping with the previous reports^29^, our results showed that pdm/BJ09 virus bound to SAα2,6 Gal linked receptor only. However, EA/FJ04 virus additionally exhibited SAα2,3 Gal linked receptor binding.

Previous studies showed that HA D222G mutation in 2009 pdm virus resulted in additional SAα2,3 linked receptor binding preference and enhanced virulence in mice and macaques^30^,^31^. EA/FJ04 virus also contains G222 in its HA protein. To determine whether residue G222 in EA HA plays a role in pathogenicity, we generated a reassortant rgEAH1/pdm virus that comprised H1 gene from EA/FJ04 virus and all other genes from pdm/BJ09 virus, which had the same genotype as Gt7 and showed binding affinity for SAα2,6 and SAα2,3 Gal linked receptors. Additionally, we introduced a HA-G222E mutation into the EA H1 gene of rgEAH1/pdm to generate a further reassortant (rgEAH1-G222E/pdm). We found that HA G222E mutation introduced into rgEAH1/pdm virus abolished binding affinity for SAα2,3 Gal linked receptor (Fig. 4). We further determined that rgEAH1/pdm virus caused 100% mortality in mice at challenge doses of 10^4^ TCID_50_ and10^5^ TCID_50_ (Fig. S4); however, rgEAH1-G222E/pdm mutant only caused slight weight loss and no mortality at the 10^4^ TCID_50_ challenge doses. Therefore, our results demonstrated that not only EA H1 gene but also HA 222G from parental EA/FJ04 virus contributed to the virulence of EA H1 reassortant virus by conferring, at least in part, affinity for the avian SAα2,3-Gal linked receptor which is mainly localized in the porcine LRT^26^. However, HA222G did not enhance the virulence of wild type EA/FJ04 in mice, suggesting that combination of HA222G with genes from pdm/BJ09 was responsible for viral virulence in mice.

### RNP of pdm/BJ09 virus conferred robust polymerase activity to reassortant viruses

**Figure 5:**
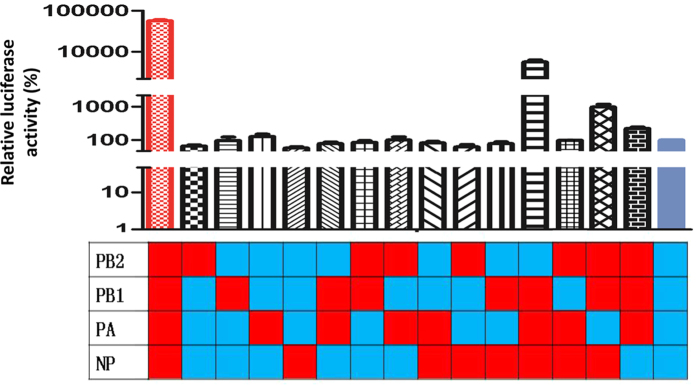
Polymerase activity of 16 RNP combinations between the EA/FJ04 and pdm/BJ09 viruses. 293T cells were transfected in duplicate with luciferase reporter plasmid p-Luci and internal control plasmid Renilla, together with different combinations of PB2, PB1, PA and NP plasmids from EA/FJ04 and pdm/BJ09 viruses. Viral segments derived from the pdm/BJ09 virus are in red, and those derived from EA/FJ04 viruses are in blue.

In the 55 reassortants, only two RNP gene combinations were found, one parental pdm/BJ09 RNP complex from the pdm/BJ09 virus and one hybrid RNP complex of PB2, PA and NP from the pdm/BJ09 virus and PB1 from EA/FJ04 virus, with a proportion of 69% (38/55) to 31% (17/55) respectively (Pearson’s χ^2^, *p* < 0.001). All reassortant viruses from pathogenicity group I and transmissibility group I housed the pdm/BJ09 virus RNP complex. Given that RNP activity correlates with replication and pathogenicity^32^,^33^, the polymerase activity of different RNP combinations between pdm/BJ09 and EA/FJ04 was evaluated by minigenome assays. Homologous parental pdm/BJ09 virus RNP complex showed significantly higher activity than that of homologous parental EA/FJ04 virus in 293T cells (Fig. 5). RNP activities of hybrid RNP complexes, however, varied substantially. All hybrid RNPs exhibited polymerase activities that were much less than the pdm/BJ09 virus RNP and more similar to the EA/FJ04 virus RNP complex. Therefore, we suggest that the selectivity for homologous RNP complex of pdm/BJ09 virus in reassortant viruses was likely due to its relatively high polymerase activity.

To evaluate the role of pdm/RNP in the pathogenicity of reassortants, a pdmRNP/EAH1N1 virus containing RNP genes from pdm/BJ09 virus and the remaining four genes from EA/FJ04 virus was generated and tested in mice. We found that the pdm/RNP genes significantly enhanced virus pathogenicity of pdmRNP/EAH1N1 in mice compared with the parental EA/FJ04 virus (Fig. S5), which suggests that pdm/RNP contributed to the virulence of reassortant virus in mice.

### Low antigenic cross-reactivity of EA SIV with human influenza vaccine strains

H1N1/pdm09-like and H3N2 antigenic components are presently incorporated in human seasonal vaccines. To assess cross-protection of such seasonal influenza vaccines against EA viruses, we performed hemagglutination inhibition (HI) assays with a panel of different sera (Table S1). The results showed that EA H1N1 viruses (Shangdong/07/2011 and Fujian/04/2007) reacted well with homologous virus sera with HI titer of 1:1280; however, they showed no reactivity with sera against human seasonal H1N1 (A/Tianjin-jinnan/15/2009, not H1N1/pdm09-like) and pdm/09 H1N1 viruses. This suggests that neither seasonal H1N1 nor pdm/09 H1N1 vaccine can currently provide effective humoral protection against EA/FJ04. Likewise, sera against the EA H1N1 viruses showed little cross-reactivity with pdm/09 H1N1 viruses of A/California/07/2009 and A/Beijing/7/2009, implying that EA H1N1 vaccine may in turn offer little protection to pdm/09 H1N1 virus infection. Notably, low cross-reactive HI antibodies against EA/FJ04 were observed in sera collected from children vaccinated by bivalent vaccine of H3N2 and H1N1 viruses, which is in accordance to previous studies^16^,^17^,^25^. Collectively, these results showed that EA H1N1 and pdm/09 H1N1, seasonal H1N1 and H3N2 viruses are antigenically distinct.

## Discussion

Eurasian avian-like swine H1N1 viruses and 2009 pandemic H1N1 viruses are prevalent in pigs in Eurasia^7^,^20^,^21^. Co-infection with two different influenza viruses in a host can mathematically produce up to 256 different reassortant viruses. In our co-infection of pigs with EA/FJ04 and pdm/BJ09 viruses, 55 out of 300 isolated virus clones were reassortants. We found that majority of the reassortant viruses (65%, 36/55) were generated from the LRT. Seventy eight % of LRT reassortants (28/36) carried the H1 HA gene from the parental EA/FJ04 virus. The 5 highly infectious reassortants genotypes of Gt2, Gt3, Gt7, Gt10 and Gt13 in guinea pigs were also amongst the most pathogenic in mice. These 5 genotypes all harbored the pdm/BJ09 RNP gene segments (PB1, PB2, PA and NP). We further demonstrated that pdm/09-RNP and EA-H1HA contributed to enhanced virulence and transmissibility in the reassortant viruses. No progeny EA/FJ04 virus was isolated which suggests that the competing pdm/BJ09 virus was much more competent at replication than EA/FJ04 virus. The present findings revealed high reassortment compatibility between EA and pdm/09 viruses in pigs, which could generate novel progeny viruses with enhanced virulence and transmissibility in mice and guinea pigs. A H3N2 variant derived from endemic H3N2 TR virus and pdm/09-origin virus in pigs has caused more than 340 human infection cases in the USA since 2011, which emphasized the role of pdm/09-origin internal gene in the genesis of influenza reassortants with the potential to infect humans^34^.

Pigs are commonly considered as “mixing vessels” for the reassortment of influenza viruses; however, how and where reassortment occurs in the pig remains unclear. Reassortment is likely to be influenced by infectious dose, cell tropism, host species and pre-existing immunity^35^. Ma *et al*. intratracheally co-infected pigs with an EA H1N1 virus and North American triple reassortant H1N1 virus. They found that reassortment mainly occurred in the lung of co-infected pigs^36^. In our study, we inoculated equal virus doses intranasally and intratracheally. Eight percent (19/240) of the isolated virus clones from the nasal passage and 60% (36/60) from the LRT were reassortants, indicating that most reassortants were isolated in the LRT.

In this study, the Gt7 reassortant viruses only containing H1 gene from EA/FJ04 showed increased pathogenicity in mice relative to either parental virus, which indicated that the EA H1 gene could be responsible for enhanced virulence in mice. Amino acid at G222 of pdm/09 HA was reported to be associated with severe disease and fatality in human patients^37^,^38^. HA- G222 variants have dual receptor specificity for SAα2,3-Gal linked and SAα2,6-Gal linked receptors, which confer expanded tissue tropism leading to increased pathogenicity in mice and ferrets^31^,^39^. We showed that a HA-G222E mutation in the EAH1/pdm reassortant resulted in significant reduction in pathogenicity in mice and eliminated its binding to SAα2,3-Gal linked receptors. However, the HA222G isoform in parental EA/FJ04 virus did not confer elevated virulence in mice, which suggested that additional contextual genes in the reassortant virus are necessary for pathogenicity. The ability of EA-H1 reassortants to bind avian SAα2,3-Gal linked receptors is expected to enhance their propagation in the porcine and human LRT where avian receptors-like are present^26^,^40^.

The other major virulence factor identified in the reassortants from co-infection with EA/FJ04 and pdm/BJ09 viruses is the dominance of the pdm/09 RNP complex. Polymerase activity of the pdm/09 RNP complex was significantly higher than those of parental EA and reassortant viruses. Collectively, the EA-H1 gene and members of pdm/09-RNP complex are major contributors to the virulence and transmissibility of EA H1 reassortants. Since recent surveillance data indicated that the internal genes of pdm/09 are widely established in pig populations in China^10^, emergence of novel reassortant viruses comprising EA-HA and pdm/09 RNP genes could pose potential threat to public health.

EA viruses have been circulating in pig populations for over 30 years. There have been occasional human cases of infection but no persistent human transmission has been documented^13^,^41^. Hence, there is little specific immunity against EA H1N1 viruses in the human population. Although, cross-reactive antibodies against EA H1N1 were found in a small proportion of human population, most do not have such antibodies^16^,^17^,^25^. We found that current seasonal H1N1 and H3N2 influenza vaccines do not appear to confer cross-reactive humoral protection against EA viruses. Therefore, there is a need of a novel EA H1N1 vaccine in anticipation of its possible emergence.

In summary, our data highlight the potential public health danger of novel viruses arising from reassortment in pigs between circulating EA swine H1N1 and pdm/09 H1N1 viruses. Such reassortants for which there is little apparent cross protection from current vaccines could be much more pathogenic and transmissible than parental viruses.

## Materials and Methods

### Ethics statement

All animal studies were performed in strict accordance with the recommendations in the Guide for the Care and Use of Laboratory Animals of China Agricultural University (CAU) (ID: SKLAB-B-2010-003) and all animal research was approved by the Beijing Association for Science and Technology of China (approval ID SYXK, Beijing, 2007–0023). Ten-day-old specific pathogen-free (SPF) chicken embryos were obtained from Merial (Beijing, China).

### Viruses and cells

Two Eurasian avian-like swine influenza viruses including A/Swine/FuJian/04/2007 (EA/FJ04, H1N1) and A/Swine/Shandong/07/2011 (H1N1), and 2009 human pandemic H1N1 viruses, A/Beijing/7/2009 (pdm/BJ09) were isolated and kept in our laboratory^42^,^43^. All viruses were grown in SPF chicken embryos. Madin-Darby canine kidney (ATCC, USA) and 293T (ATCC, USA) cells were cultured in high glucose Dulbecco’s modified Eagle medium (DMEM) supplemented with antibiotics (100 U/ml penicillin and 100 μg/ml streptomycin) and 10% fetal bovine serum (Invitrogen) at 37 °C with 5% CO_2_.

### Co-infection of pigs and samples collection

Four to 5 weeks old Landrace-Large White cross pigs were sourced from a high health status farm. All animals were sero-negative for circulating influenza viruses (H1N1, H3N2, H5N1 and H9N2) by Hemagglutination inhibition (HI) assay. Six pigs were inoculated intranasally and equally intratracheally with 10^6.0^ TCID_50_ of EA/FJ04 and pdm/BJ09 in 3 ml phosphate buffered saline (1.5mL for each challenge route). All inoculated pigs were observed twice daily for clinical signs until the end of the experiment. Nasal washes were collected from all pigs every 24 h for 5 days post infection (dpi). Three pigs were euthanized at 3 and 5 dpi. The lungs were flushed with 50 ml fresh DMEM to obtain bronchoalveolar fluid (BALF) from each euthanized pig.

### Virus plaque purification and identification of the genetic component

Each BALF sample and nasal wash from co-infected pigs was serially diluted tenfold and inoculated onto a monolayer of MDCK cells grown in six-well plates. Single plaques were randomly selected (approx. 10 plaques per sample) and plaque purification was performed three rounds in order to purify the virus. Viral RNA was extracted from purified virus by using the QIAamp Viral RNA Minikit (Qiagen), and RT-PCR was performed to amplify the segments of influenza A virus as described previously^44^. Full genomic DNA was sequenced by Tingske Biological Technology Co. Ltd (Beijing, China). To identify the reassortant patterns of progeny virus, all sequence results were analyzed by using DNA star software (Lasergene 7.1).

### Receptor binding assays

Receptor binding specificity was analyzed by a solid-phase direct binding assay of biotinylated sialylglycopolymers as previously described^29^. Sodium salts of sialylglycopolymers poly-l-glutamic acid backbones containing N-acetylneuraminic acid linked to galactose through either an α-2,3 [Neu5Acα2,3Galβ1,4GlcNAcβ1-pAP] or an α-2,6 [Neu5Acα2,6Galβ1,4GlcNAcβ1-pAP] were kindly provided by the Glycotech, Gaithersburg, MD

### Ribonucleoprotein (RNP) minigenome luciferase assay

RNP genes (PB2, PB1, PA and NP) from EA/FJ04 and pdm/BJ09 viruses were cloned into pcDNA3.1 expressing vector. Polymerase activity of RNP from different gene combination of EA/FJ04 and pdm/BJ09 viruses was assayed in 293T cells as previously described^45^. Renilla luciferase plasmid pRL-TK (Promega) was also co-transfected to normalize the transfection efficiency. Firefly luciferase expression was normalized to Renilla luciferase expression (relative activity). The relative activity of the EA/FJ04 RNP was set as 100%, and the relative activities of the other reassortant RNP were presented relative to that.

### Reverse genetics

All segments of EA/FJ04 and pdm/BJ09 were cloned into dual-promoter plasmid PHW2000 as described previously^29^; all plasmids were verified by sequencing to preclude any mutations. A pdmRNP/EAH1N1 reassortant virus containing RNP of pdm/BJ09 and other four genes from EA/FJ04 was generated by co-transfecting 293T cells with plasmids of PHW2000-EA/FJ04-HA, NA, M, NS and PHW2000-pdm/BJ09-PB2, PB1, PA, NP. Similarly, a reassortant virus rgEAH1/pdm with HA gene from EA/FJ04 and the other seven genes from pdm/BJ09 was generated. To generate rgEAH1-G222E/pdm virus that contains a G to E substitution at residue 222 of HA, HA-G222E was introduced into PHW2000-EA/FJ04-HA by site-directed mutagenesis kit (Qiagen). All viruses were propagated in MDCK cells and sequence verified prior to animal studies.

### Mouse experiments

To evaluate the virulence of all reassortant viruses, groups of nineteen 5-week-old female BALB/c mice were intranasally inoculated with 50 μL of 10-fold serial dilutions of each virus in PBS. Three mice infected with 10^5^ TCID_50_ virus from each group were euthanized at 4 dpi and lung tissues were harvested for virus titration and hematoxylin and eosin staining. Body weights of all mice were monitored daily for 14 dpi, and mice that lost more than 25% of their body weight were euthanized on humane ground. LD_50_ were calculated and expressed as the TCID_50_ value corresponding to 1 LD_50_.

In order to examine the roles of EA/FJ04 H1-222G and pdm/BJ09 RNP genes in the pathogenicity of reassortant viruses, groups of six mice were intranasally inoculated with rgEAH1-G222E/pdm, rgEAH1/pdm, pdmRNP/EAH1N1 and EA/FJ04 at two infection doses of 10^4^ and 10^5^ TCID_50_, weight loss and survival rate were monitored daily until the end of this study (14dpi).

### Guinea pig experiment

Female SPF/VAF guinea pigs (*Hartley strain*, 300–350 g, Vital River Laboratory Animal Technology), serum-negative for influenza virus, were used in these studies. Animals were anesthetized by muscular injection with zoletil 100 (tiletamine-zolazepam; Virbac S.A., Garros, France) (10–15 mg/kg). Groups of three animals were intranasally inoculated with 10^5^ TCID_50_ of test viruses and housed in a cage per group. At 24 h post infection, three naïve guinea pigs were placed in the same cage and co-housed in this way for a total of 14 days. On 2, 4, 6, and 8 dpi, nasal washes of the three inoculated animals and three contact animals were collected and titrated. The area under the nasal wash curve between days 2 and 6 post inoculation (AUC_2–6 dpi_) was calculated from the virus titers of nasal wash of all guinea pig using GraphPad Prism 6 software as previously described^27^. All animals were euthanized at 14 dpi and sera were collected for seroconversion tests.

### HI assay and antisera

HI assays were used to determine the antigenic cross-reactivity of EA SIV with prevailing human seasonal H1N1, human seasonal H3N2, and pandemic H1N1/2009 viruses. Positive human sera were collected from two 4-year-old children at 30 days after two immunizations with influenza bivalent vaccine comprising A/Victoria/361/2009 and A/California/07/2009 by Chinese National Influenza Center. HI antigens of A/Victoria/361/2009 (human seasonal H3N2), A/Tianjin-jinnan/15/2009 (human seasonal H1N1), A/California/07/09 (pandemic 2009 H1N1), and hyperimmune goat serum against human seasonal A/Tianjin-jinnan/15/2009 were provided by Chinese National Influenza Center. Antisera against pdm/BJ09 or EA/FJ04 were prepared from ferrets inoculated by 10^6^ TCID_50_ of A/Beijing/7/2009 or A/Swine/Fujian/04/2007. HI tests were performed in accordance with WHO guidelines. All HI assays were performed in duplicates.

### Statistical analysis

Statistical analysis and graphical presentation were performed using the GraphPad Prism 6 software. Viral titers were calculated as medians with standard deviations. Statistical significance (P values) between the groups was compared using one-way analysis of variance (ANOVA) and Tukey’s multiple comparison tests.

## Additional Information

**How to cite this article**: Kong, W. *et al*. Transmission and pathogenicity of novel reassortants derived from Eurasian avian-like and 2009 pandemic H1N1 influenza viruses in mice and guinea pigs. *Sci. Rep*. **6**, 27067; doi: 10.1038/srep27067 (2016).

## Supplementary Material

Supplementary Information

## Figures and Tables

**Table 1:** Genotypes and distribution of reassortants recovered from coinfected pigs.

	Virus Segments								Lung^a^	N.S.^b^	Percentage
	PB2	PB1	PA	HA	NP	NA	M	NS			
Gt1	P	P	P	E	P	P	E	P	1/60	0/240	1/300 (0.3%)
Gt2	P	P	P	E	P	P	P	E	14/60	0/240	14/300 (4.7%)
Gt3	P	P	P	E	P	E	E	E	1/60	0/240	1/300 (0.3%)
Gt4	P	E	P	E	P	E	P	E	2/60	0/240	2/300 (0.7%)
Gt5	P	P	P	E	P	E	P	P	1/60	0/240	1/300 (0.3%)
Gt6	P	E	P	E	P	P	P	E	2/60	0/240	2/300 (0.7%)
Gt7	P	P	P	E	P	P	P	P	4/60	1/240	5/300 (1.7%)
Gt8	P	E	P	E	P	P	E	E	2/60	0/240	2/300 (0.7%)
Gt9	P	E	P	E	P	E	E	P	3/60	0/240	3/300 (1.0%)
Gt10	P	P	P	E	P	E	P	E	0/60	2/240	2/300 (0.7%)
Gt11	P	E	P	P	P	P	P	E	2/60	0/240	2/300 (0.7%)
Gt12	P	E	P	P	P	E	P	E	1/60	0/240	1/300 (0.3%)
Gt13	P	P	P	P	P	P	P	E	0/60	7/240	7/300 (2.3%)
Gt14	P	P	P	P	P	E	P	P	0/60	7/240	7/300 (2.3%)
Gt15	P	E	P	P	P	P	P	P	0/60	2/240	2/300 (0.7%)
Gt16	P	E	P	P	P	P	E	E	1/60	0/240	1/300 (0.3%)
Gt17	P	E	P	P	P	E	E	E	2/60	0/240	2/300 (0.7%)
pdm/BJ09	P	P	P	P	P	P	P	P	24/60	221/240	245/300 (83%)
EA/FJ04	E	E	E	E	E	E	E	E	0/60	0/240	0/300 (0%)

The virus segments derived from the pdm/BJ09 are denoted P, and those derived from EA/FJ04 virus are denoted E.

^a^No. of reassortant isolates/total no. of viruses examined in BALF.

^b^No. of reassortant isolates/total no. of viruses examined in nasal swab specimen, N.S. nasal swab.

**Table 2:** Pathogenicity of reassortant viruses in mice compared with parental EA/FJ04 and pdm/BJ09 viruses.

	Genotype^a^	Virus Segments								LD50 (Log_10_pfu)	MST^b^ (Days)	Viral titer^c^ (TCID_50_/mL)	Nature^d^
		PB2	PB1	PA	HA	NP	NA	M	NS				
Pathogenicity group III	Gt1	P	P	P	E	P	P	E	P	≥5.5	≥14	5.3 ± 0.4	
	Gt9	P	E	P	E	P	E	E	P	≥5.5	≥14	5.5 ± 0.1	
	Gt12	P	E	P	P	P	E	P	E	≥5.5	≥14	5.4 ± 0.1	
	EA/FJ04	E	E	E	E	E	E	E	E	≥5.5	≥14	3.8 ± 0.4	
Pathogenicity group II	pdm/BJ09	P	P	P	P	P	P	P	P	4.5	4.3	4.9 ± 0.5	
	Gt4	P	E	P	E	P	E	P	E	4.5	4.3	6.1 ± 0.3	F^10^
	Gt6	P	E	P	E	P	P	P	E	4.3	4	5.8 ± 0.6	
	Gt8	P	E	P	E	P	P	E	E	4.3	4	5.3 ± 0.4	
	Gt11	P	E	P	P	P	P	P	E	4.5	4.7	5.8 ± 0.4	
	Gt14	P	P	P	P	P	E	P	P	4.3	4.7	5.3 ± 0.1	F^20,23^
	Gt15	P	E	P	P	P	P	P	P	4.5	8.3	5.3 ± 0.4	
	Gt16	P	E	P	P	P	P	E	E	4.5	6.3	5.7 ± 1.1	
	Gt17	P	E	P	P	P	E	E	E	4.5	4.7	6.4 ± 0.1	
Pathogenicity group I	Gt2	P	P	P	E	P	P	P	E	3.8	4.7	5.7 ± 0.1	
	Gt3	P	P	P	E	P	E	E	E	3.8	4.7	5.3 ± 0.1	
	Gt5	P	P	P	E	P	E	P	P	3.8	4.3	5.7 ± 0.1	F^10,22,23^
	Gt7	P	P	P	E	P	P	P	P	3.5	4.7	6 ± 0.7	
	Gt10	P	P	P	E	P	E	P	E	3.5	5.3	5.1 ± 0.3	
	Gt13	P	P	P	P	P	P	P	E	3.5	4	4.9 ± 0.5	

^a^Pathogenicity group I: reassortants with higher pathogenicity than both parental viruses; Pathogenicity group II: reassortants with pathogenicity similar to that of the pdm/BJ09 virus; Pathogenicity group III: reassortants with pathogenicity similar to or lower than that of the EA/FJ04 virus. The virus segments derived from the pdm/BJ09 are denoted P, and those derived from EA/FJ04 virus are denoted E.

^b^MST, mean survival time of mice infected with 10^5^TCID_50_

^c^Virus titers in lung are means ± SDs of the log10 TCID_50_/mL at 4 dpi of three mice infected with 10^5^TCID_50_.

^d^If reassortant virus was isolated from pigs in field, it is noted as F. References were added.
