# Supplementary material for: Transmission and pathogenicity of novel reassortants derived from Eurasian avian-like and 2009 pandemic H1N1 influenza viruses in mice and guinea pigs
Source: Sci Rep. 2016 Jun 2;6:27067. doi: 10.1038/srep27067 (PMC4890009; doi:10.1038/srep27067)
Supplement: Supplementary Information [file srep27067-s1.docx]

# Transmission and pathogenicity of novel reassortants derived from Eurasian avian-like and 2009 pandemic H1N1 influenza viruses in mice and guinea pigs

Weili Kong^1,#^,Qinfang Liu^2,#^,Yipeng Sun^1^, Yu Wang^1^, Huijie Gao^1^, Lirong Liu^1^, Zhihua Qin^1^, Qiming He^1^, Honglei Sun^1^, Juan Pu^1^, Dayan Wang^3^, Xin Guo^1^, Hanchun Yang^1^, Kin-Chow Chang^4^, Yuelong Shu^3^, and Jinhua Liu^1,§^

Author Affiliation:

^1^Key Laboratory of Animal Epidemiology and Zoonosis, Ministry of Agriculture, College of Veterinary Medicine, and State Key Laboratory of Agrobiotechnology, China Agricultural University, Beijing, 100193,China

^2^Department of Avian Infectious Disease, Shanghai Veterinary Research Institute, ChineseAcademy of Agricultural Sciences, Shanghai, Innovation Team for Pathogen Ecology Research on Animal Influenza Virus, Shanghai, 200241 China

^3^National Institute for Viral Disease Control and Prevention, China CDC, Key Laboratory for Medical Virology, National Health and Family Planning Commission, Beijing 102206, China

^4^School of Veterinary Medicine and Science, University of Nottingham, Sutton Bonington Campus, Loughborough, LE12 5RD ,United Kingdom

^#^W.K., and Q.L. contributed equally to this work.

^§^To whom correspondence should be addressed. Email: ljh@cau.edu.cn

**Supplementary Data**

**Fig. S1**


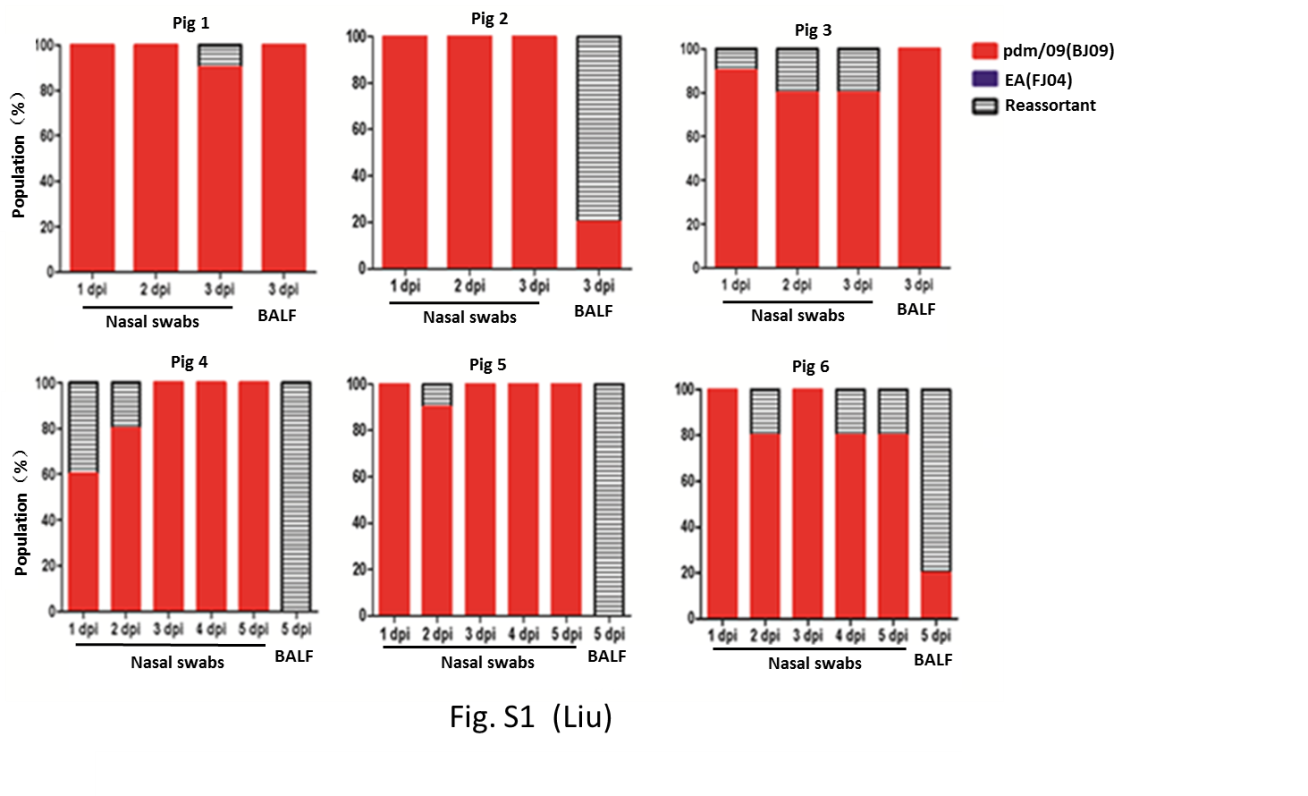


Fig. S1. Reassortant prevalence from six pigs co-infected with pdm/BJ09 and EA/FJ04 viruses. Percentage of pdm/BJ09, EA/FJ04 and reassortant genotypes of all viruses isolated from nasal swabs and BALF of each pig at different days post infection.

.

**Fig. S2**


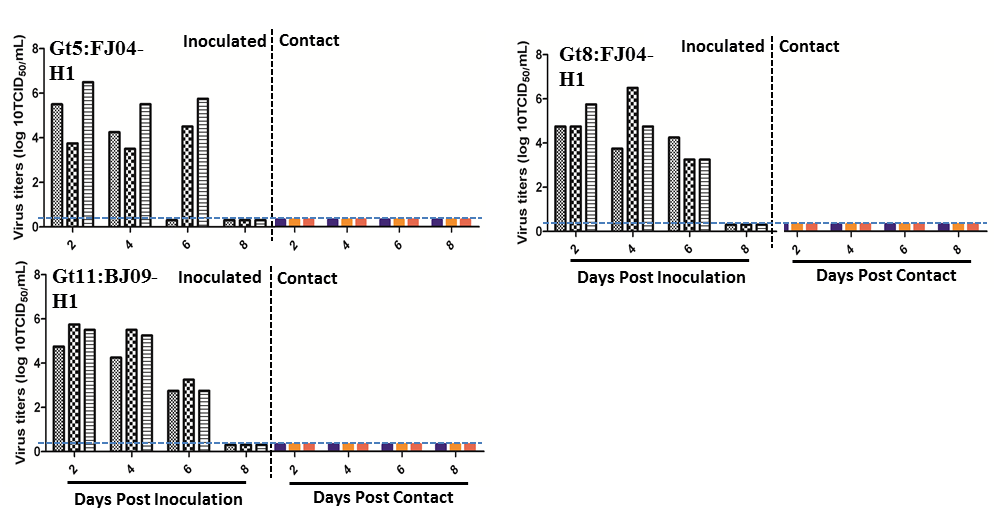


Fig. S2. Replication and transmission of reassortants with transmissibility similar to parental EA/FJ04 virus in guinea pigs (transmission group III). Each group of three guinea pigs (grey columns) was i.n. inoculated with 10^5^TCID_50_ of each virus (Gt5, 8 and 11); 24 hours later three in-contact guinea pigs (colorful columns) were placed with each group. Nasal washes were collected every two days from all animals. There was no viral transmission to in-contact animals with each of the three viruses. Each bar represents the virus titer from nasal wash of an individual animal. The dashed blue lines indicate the lower limit of detection.

**Fig. S3**


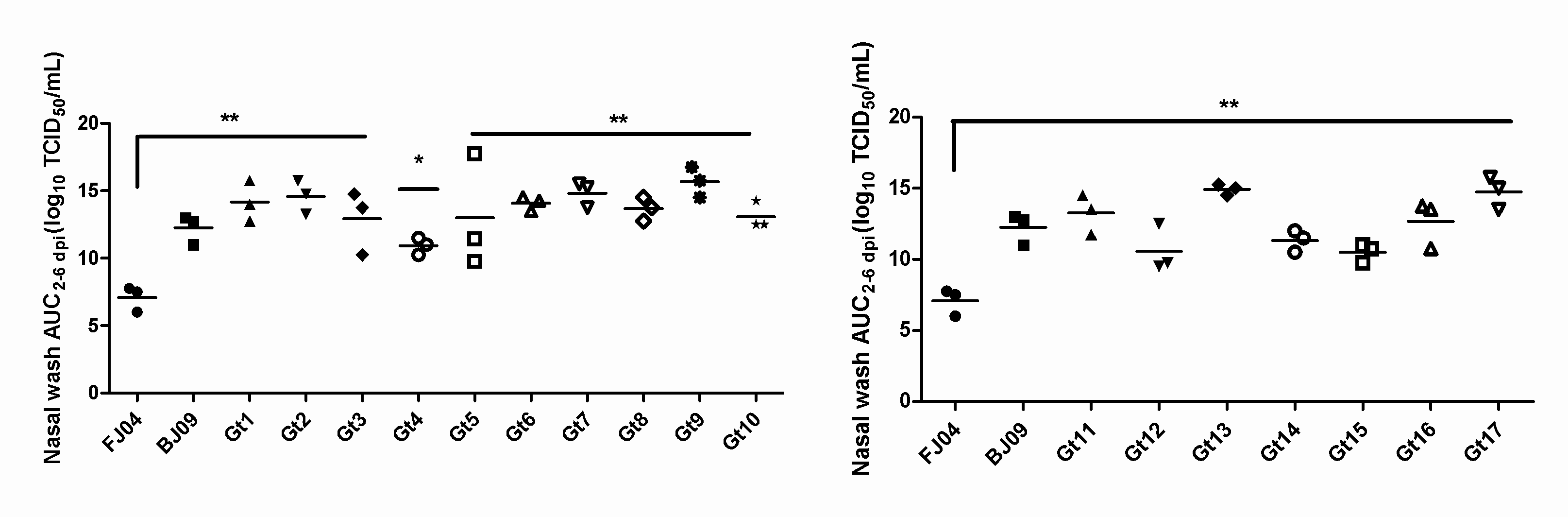


Fig. S3. The mean nasal wash AUC_2–6 dpi_values for guinea pigs inoculated with reassortants and parental viruses. Horizontal lines denote the mean AUC_2–6 dpi_. *, *P* < 0.05; **, *P* < 0.01.

**Fig. S4**


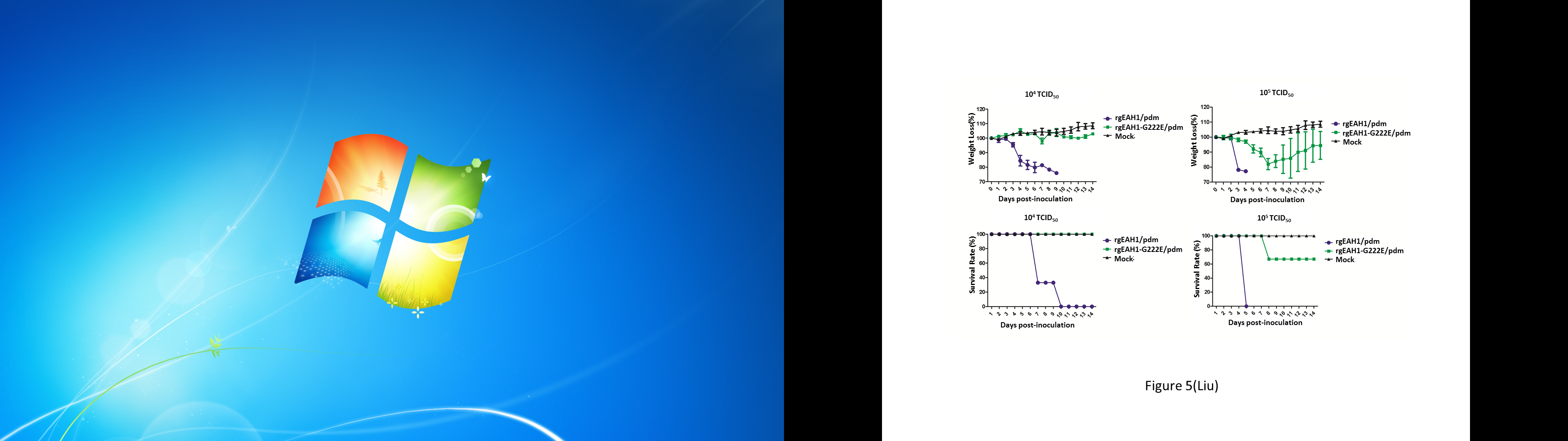


Fig. S4 . Survival rate and weight loss in mice infected with reassortant rgEAH1/pdm and its mutant rgEAH1-G222E/pdm. Loss in body weight was determined daily up to 14 days post infection with different infection doses of each virus.

**Fig. S5**


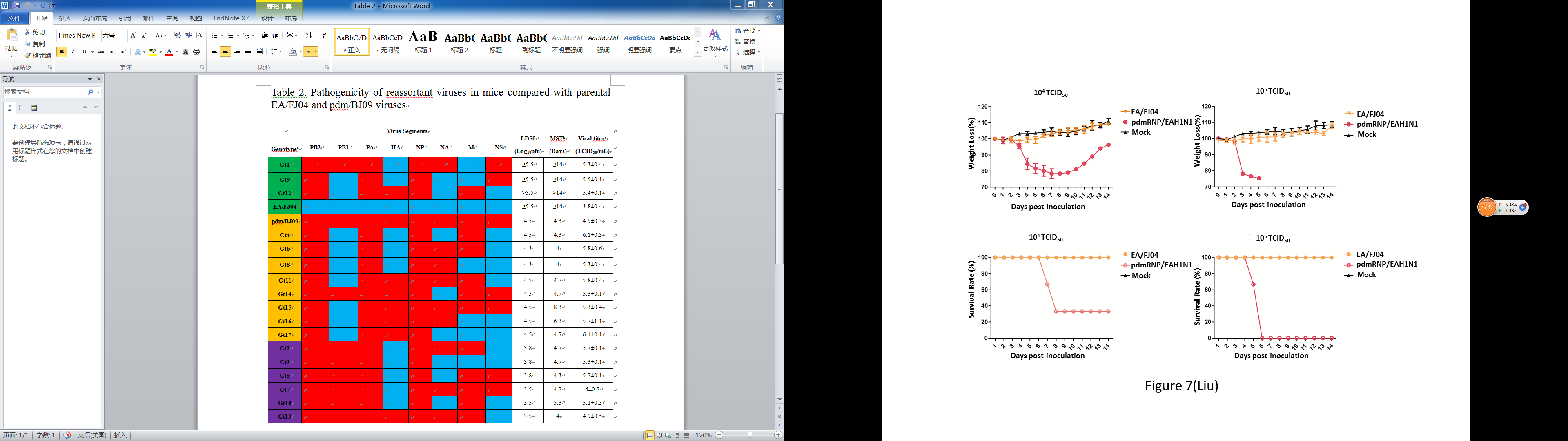


Fig.S5. Survival rate and weight loss in mice infected with reassortant pdmRNP/EAH1N1and parental EA/FJ04 viruses. Loss in body weight was determined daily up to 14 days post infection at different infection doses with each virus.

Table S1. Antigenic analysis of swine and human influenza viruses

| Virus | HI titers to antiserum | | | | |
| --- | --- | --- | --- | --- | --- |
|  | Human sera against Pdm/09 H1N1+ seasonal H3N2 vaccine † | | Goat serum against seasonal  H1N1 | Ferret serum against pdm/BJ09 | Ferret serum against EA/FJ04 |
|  | 1 | 2 |  |  |  |
| A/California/07/2009(H1N1) | **2560** | **640** | - | 1280 | 40 |
| A/Beijing/7/2009(H1N1) | 2560 | 640 | - | **1280** | 40 |
| A/Victoria/361/2009(H3N2) | **320** | **320** | - | - | - |
| A/Tianjin-jinnan/15/2009(H1N1) | 80 | 40 | **1024** | - | - |
| A/Swine/Shandong/07/2011(H1N1) | 40 | 20 | - | - | 1280 |
| A/Swine/Fujian/04/2007 (H1N1) | 40 | 20 | - | - | **1280** |

†:Antisera were obtained from two 4-year-old children who were vaccinated with human influenza bivalent vaccine derived from A/Victoria/361/2009(H3N2) and A/California/07/2009(H1N1).

Homologous titers are shown in boldface.

-: HI <10
